# Protease-Activated Receptor F2R Is a Potential Target for New Diagnostic/Prognostic and Treatment Applications for Patients with Ovarian Cancer

**DOI:** 10.3390/ijms26178529

**Published:** 2025-09-02

**Authors:** Riya Khetan, Noor A. Lokman, Preethi Eldi, Zoe K. Price, Martin K. Oehler, Doug A. Brooks, Anton Blencowe, Sanjay Garg, Carmela Ricciardelli, Hugo Albrecht

**Affiliations:** 1 Centre of Pharmaceutical Innovation, UniSA Clinical and Health Sciences, University of South Australia, Adelaide, SA 5000, Australia; riya.khetan@mymail.unisa.edu.au (R.K.); anton.blencowe@unisa.edu.au (A.B.); sanjay.garg@unisa.edu.au (S.G.); 2 Discipline of Obstetrics and Gynaecology, Adelaide Medical School, Robinson Research Institute, University of Adelaide, Adelaide, SA 5000, Australia; noor.lokman@adelaide.edu.au (N.A.L.); zoe.price@adelaide.edu.au (Z.K.P.); martin.oehler@adelaide.edu.au (M.K.O.); 3 UniSA Clinical and Health Sciences, University of South Australia, Adelaide, SA 5000, Australia; preethi.eldi@unisa.edu.au (P.E.); doug.brooks@unisa.edu.au (D.A.B.); 4 Department of Gynaecological Oncology, Royal Adelaide Hospital, Adelaide, SA 5000, Australia; 5 Applied Chemistry and Translational Biomaterials Group, Centre of Pharmaceutical Innovation, UniSA Clinical and Health Sciences, University of South Australia, Adelaide, SA 5000, Australia

**Keywords:** F2R/PAR1, diagnostic/prognostic, biomarker, therapeutic target, ovarian cancer

## Abstract

Effective treatment of ovarian cancer is limited by late-stage detection and chemotherapy resistance. There is a clinical need for the discovery of novel molecular targets to enable the development of innovative theranostic approaches. We investigated the coagulation factor II receptor/protease-activated receptor 1 (F2R/PAR1) as a potential diagnostic/prognostic biomarker and therapeutic target for ovarian cancer treatment. Public RNA sequence and DNA microarray data were used to analyze F2R gene expression in ovarian cancers, with protein expression confirmed in tumor samples by flow cytometry, immunofluorescence, and immunohistochemistry (IHC). Functional assays were conducted to study effects of F2R suppression on tumor progression. Our analysis confirmed elevated F2R mRNA and protein expression in ovarian cancers, notably in patients with metastatic and chemotherapy-resistant disease. Kaplan–Meier survival analysis demonstrated an association between high F2R protein detection and reduced progression-free survival. F2R suppression in ovarian cancer cell lines reduced tumor cell motility, invasion, spheroid formation, and metabolism and enhanced carboplatin sensitivity. F2R is a compelling diagnostic/prognostic and therapeutic target that could be used to treat chemotherapy-resistant and metastatic disease. The evaluation of novel F2R targeting strategies, using antibody-conjugated drugs or F2R ligand-decorated drug carriers, could lead to the development of effective therapeutics for patients with ovarian cancer.

## 1. Introduction

Ovarian cancer is among the most lethal gynecological tumors. Late-stage diagnosis, chemotherapy resistance, and frequent clinical recurrence directly contribute to poor patient outcomes [1]. Ovarian cancer can be stratified into five major histological subtypes [2], most of which manifest as poor prognosis associated with rapid disease progression and high rates of metastasis. There is an urgent clinical need for improved diagnostic/prognostic technologies and effective treatment options by, for example, exploiting newly identified actionable molecular targets.

We previously identified significant overexpression of G protein-coupled receptors (GPCRs) in ovarian cancer tissues, including the protease-activated receptor 1 (PAR1), also known as coagulation factor II thrombin receptor (F2R) [3]. Protease-activated receptors (PARs) play a pivotal role in cancer progression, particularly in processes linked to invasion and metastasis [4,5,6]. F2R is activated by serine proteases, predominantly thrombin, which cleaves its N-terminal domain to generate a tethered ligand. Downstream signaling of F2R is strongly linked to inflammation and coagulation, two processes intricately involved in cancer progression [7]. Furthermore, F2R has been implicated in several cancer hallmarks, including cell migration, invasion, and proliferation, across several cancer types, including melanoma and gastric cancer [8,9,10,11]. F2R is therefore a potential molecular target to further investigate its expression and functional role in ovarian cancer.

There is significant evidence for the involvement of F2R in the biology of other cancers. In triple-negative breast cancer, F2R is known to drive tumor metastasis and invasion by promoting the secretion of matrix metalloproteinases (MMPs) [12]. Similarly, in gastric cancers, F2R has been associated with angiogenesis via the activation of the Ras-associated protein 1 and mitogen-activated protein kinase (Rap1/MAPK) pathways, which contribute to tumor growth [13]. In melanomas, a link to enhanced cell motility and metastasis via the activation of the extracellular signal-regulated kinase (ERK) signaling pathway has been established [14]. Despite these well-known multifaceted roles of F2R in other cancers, its role in ovarian cancer remains inadequately explored [15,16,17,18,19].

We previously observed increased F2R expression in ovarian cancer [3], prompting us to further investigate its clinical significance using public datasets. We confirmed *F2R* overexpression in ovarian cancer compared to healthy tissues. F2R protein expression was also confirmed in patient-derived samples using flow cytometry, immunofluorescence, and IHC analyses. Kaplan–Meier survival analysis revealed a significant association between elevated F2R protein levels and poor prognosis. Functional in vitro studies using *F2R* gene knockdown in ovarian cancer cell lines significantly impaired key oncogenic processes, including cellular invasion, motility, and spheroid formation, while also sensitizing cells to carboplatin treatment. Our findings highlight that F2R is a promising biomarker for disease detection and a target for therapeutic intervention, which may be used to overcome the current treatment limitations associated with drug resistance and metastasis.

## 2. Results

### 2.1. F2R Gene Expression Levels Were Elevated in Ovarian Cancers

**Figure 1:**
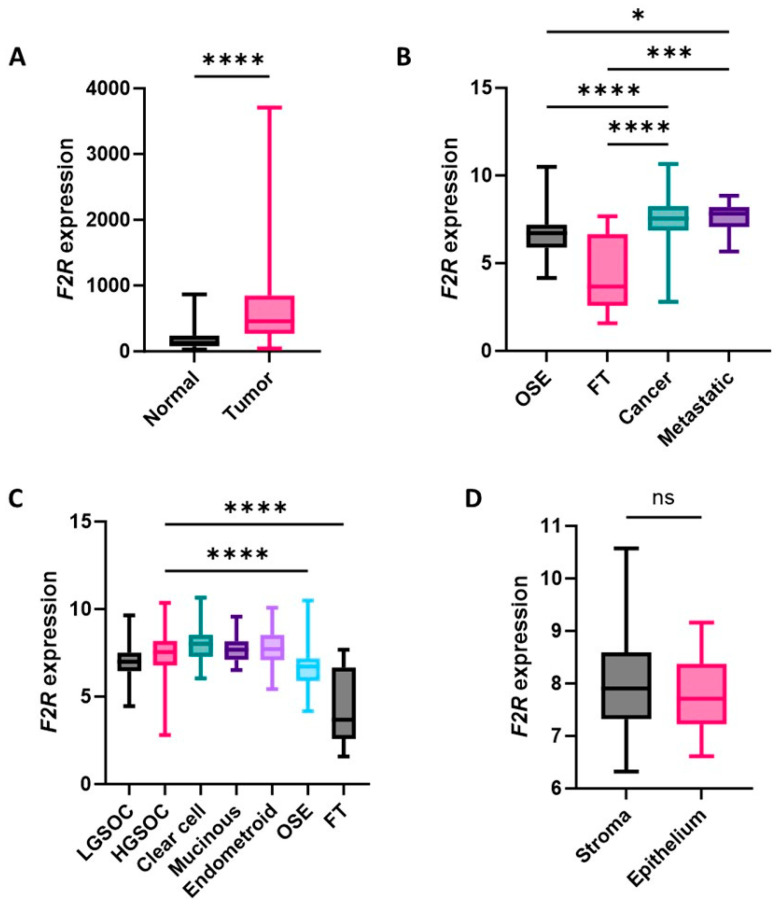
Box plots showing quartile distributions of *F2R* gene expression in ovarian cancer and non-cancer tissues. (**A**) *F2R* expression (TNMplot, RNA sequence data) in normal (*n* = 133) and serous carcinoma (*n* = 374) tissues. (**B**) *F2R* expression (GENT2) in the ovarian surface epithelium (OSE) (*n* = 66), fallopian tube (FT) (*n* = 40), all epithelial ovarian tumor subtypes (*n* = 1162), and metastatic tumor (*n* = 10). (**C**) *F2R* expression (GENT2) in ovarian cancer subtypes, including low-grade serous ovarian cancer (LGSOC) (*n* = 41), high-grade serous ovarian cancer (HGSOC) (*n* = 806), clear-cell ovarian cancer (*n* = 77), mucinous ovarian cancer (*n* = 32), and endometroid ovarian cancer (*n* = 98), compared to OSE and FT. (**D**) *F2R* expression (GSE40595) in the stroma and epithelium of laser micro-dissected HGSOC tissues from matching patients (*n* = 28). Statistical analysis included Kruskal–Wallis and Dunn’s multiple comparison test (**A**,**B**), Mann–Whitney test (**C**), and Wilcoxon test (**D**); **** *p* < 0.0001, *** *p* < 0.001, * *p* < 0.05, and non-significant (ns).

Public RNA sequence data were accessed via the TNMplot database to compare F2R gene expression in serous ovarian carcinoma and healthy tissue samples [20,21]. F2R gene expression was significantly increased in ovarian cancer compared to normal tissues (*p* < 0.0001; Figure 1A), indicating that F2R is a potential diagnostic and anti-cancer target. In addition, mRNA microarray data were analyzed from the GENT2 database, which included OSE and FT as normal tissue controls, as well as primary and metastatic ovarian cancer tissues. Significantly higher F2R gene expression was detected in ovarian cancer and metastatic tissue samples when compared to both OSE and FT control samples (Figure 1B).

To gain more detailed information, the cancer samples were stratified according to different ovarian cancer subtypes, including LGSOC, HGSOC, clear-cell, mucinous, and endometroid cancers, revealing significantly elevated *F2R* expression for all subtypes (*p* < 0.05, Figure 1C) and *p* < 0.0001 for the most prevalent subtype, HGSOC. No significant difference in F2R gene expression was observed between the stroma and epithelium of matching HGSOC tissues (Figure 1D). Overall, these findings indicate that the F2R may be a suitable tumor marker across different ovarian cancer subtypes, with specificity for cancer compared to benign and normal ovarian tissue.

### 2.2. F2R Protein Was Expressed in Ascites-Derived Ovarian Cancer Cells

**Figure 2:**
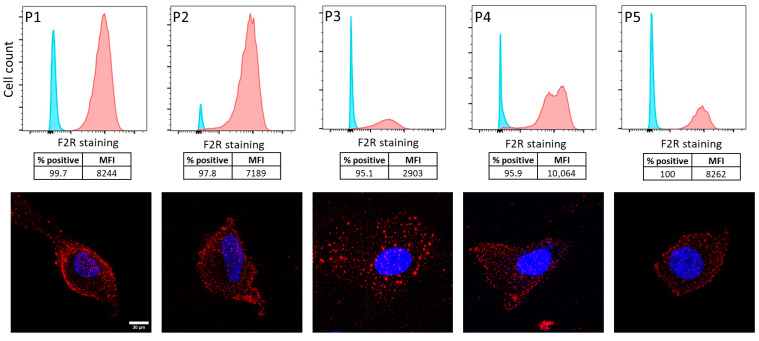
F2R protein expression in ascites-derived ovarian cancer cell samples. F2R protein expression was observed in five patient-derived ascites samples (P1–P5) using flow cytometry (**top panel**, *x*-axis and *y*-axis representing F2R staining and count, respectively) and representative immunofluorescence images (**bottom panel**). Flow cytometry histogram represents no stain control (blue) and positive F2R staining (red). Positive cells (%) and median fluorescent intensity (MFI) are provided for each flow cytometry run. Immunofluorescence images display F2R protein expression (red) and nuclei stained with Hoechst stain (blue) for representative cells from patient samples (P1–P5); scale bar in bottom-left corner = 100 µm.

Whilst protein expression depends on mRNA transcription, there is often not a direct correlation between gene and protein expression [22], hence the need to investigate F2R protein expression in patient-derived tumor samples. For this purpose, intracellular F2R expression was first analyzed in ascites-derived ovarian cancer cell samples from five patients with HGSOC using flow cytometry and immunofluorescence (Figure 2). Clear shifts in fluorescence intensity between the control (blue peaks indicating no antibody staining) and F2R-positive populations (red peaks indicating F2R staining) were observed in all five primary ovarian cancer samples, confirming the presence of F2R protein in primary HGSOC.

F2R expression in these patient-derived ascites cell samples was also confirmed using immunofluorescence imaging using surface staining (red fluorescence), and the cells were co-stained with Hoechst stain (blue, indicating nucleus). Flow cytometry provided a quantitative assessment of F2R-positive populations, while immunofluorescence offered insights of F2R expression at the cellular level. The immunofluorescence micrographs represented labeling of live cells, with evidence of labeling at or in close proximity to the cell surface and in structures that resembled endosome vesicles, which would be consistent with cell surface labeling followed by endocytosis.

### 2.3. F2R Protein Expression Was Elevated in Ovarian Cancer Compared to Normal Tissues

**Figure 3:**
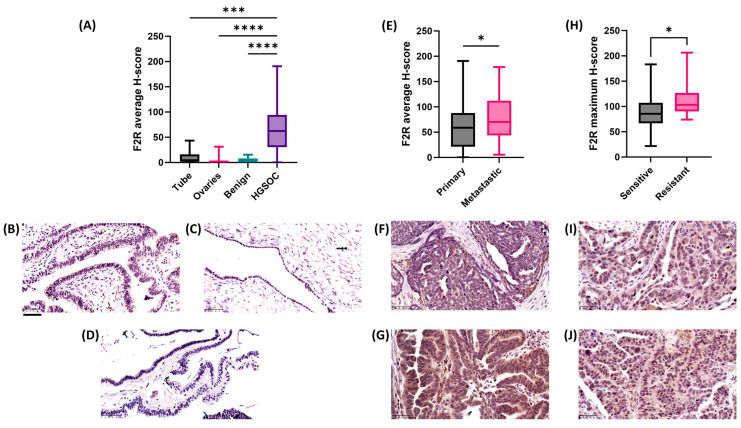
F2R protein expression in ovarian cancer tissues. (**A**) F2R protein (average H-score) in normal fallopian tube epithelial cells (*n* = 11), ovarian surface epithelium (*n* = 10), and benign ovarian tumors (*n* = 10) compared to HGSOCs (*n* = 118, primary and metastatic). (**B**–**D**) Representative figures for normal tube, ovary, and benign tumor samples, respectively. (**E**) Average H-score F2R staining in primary (*n* = 83) and metastatic (*n* = 35) HGSOC tissues. (**F**,**G**) Representative image of F2R immunostaining in primary and metastatic HGSOC tissue, respectively. (**H**) Maximum H-score F2R staining in the tumor region of chemotherapy-sensitive (*n* = 19) and -resistant (*n* = 19) HGSOC tissues. (**I**,**J**) Representative image of F2R immunostaining in sensitive and resistant HGSOC tissues, respectively. Statistical analysis included Kruskal–Wallis and Dunn’s multiple comparison test (**A**) and Mann–Whitney test (**E**,**H**) (**** *p* < 0.0001, *** *p* < 0.001, and * *p* < 0.05). All IHC images are at equal scale, with individual 50 µm bars shown in bottom-left corners. For better clarity, an enhanced 50 µm scale bar has been added as a solid black line below panel (**B**).

We investigated the tissue expression of F2R using a larger collection of histological specimens prepared from normal FT (*n* = 11), normal ovaries (*n* = 12), benign ovarian tumors (*n* = 9), and HGSOCs (*n* = 118) and assessed F2R protein by IHC. At this stage, we selected HGSOC, as this is the most common ovarian cancer subtype, and a relatively large patient group including matching data, such as treatment sensitivity and survival, were available for this study. There was a good reason to speculate that other ovarian cancer subtypes might show increased F2R protein, since we showed elevated gene expression across all the stratified cohorts (Figure 1C). Our IHC experiments revealed significantly elevated F2R protein in HGSOC tissues compared to normal ovaries, FT, and benign ovarian tissue (Figure 3A). Representative IHC images of normal FT (Figure 3B), ovary (Figure 3C), and benign ovarian tissues (Figure 3D) demonstrated only minimal F2R staining.

The comparison of F2R expression between primary and metastatic tumor tissues revealed significantly higher F2R protein expression in metastatic HGSOC tissues (Figure 3E, *p* = 0.0251). Representative images also confirmed stronger F2R labeling (*p* > 0.05) in metastatic tissue (Figure 3G) compared to primary cancer tissue (Figure 3F). This finding suggested that F2R could play an important role in ovarian tumor progression and metastasis.

Most interestingly, for the F2R protein expression in chemotherapy-sensitive versus chemotherapy-resistant HGSOC tissues, the maximum F2R H-score revealed the chemotherapy-resistant ovarian cancer samples to exhibit higher F2R protein levels compared to cancers from chemotherapy-sensitive HGSOC patients (Figure 3H–J), indicating that F2R could have prognostic potential as a chemotherapy resistance marker and also be a target for future theranostic strategies.

### 2.4. High F2R Protein Expression in HGSOC Patients Was Linked to Poor Patient Outcome

**Figure 4:**
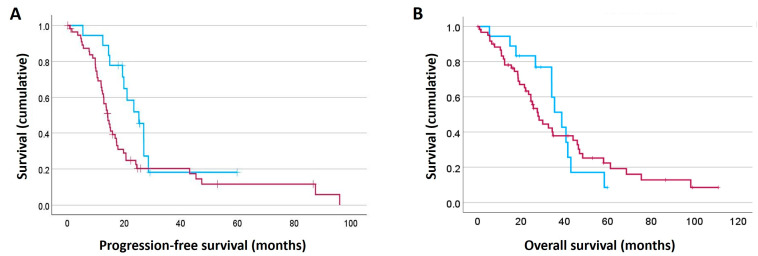
Survival analysis relative to high vs. low F2R protein expression in tumor samples from patients with ovarian cancer. Kaplan–Meier plots showing (**A**) progression-free survival (*n* = 75, *p* = 0.037) and (**B**) overall survival (*n* = 81, *p* = 0.418) for HGSOC patients stratified based on F2R H-scores into low (lowest 25% quartile) and high (remaining 75% of samples) expressers. Red lines indicate high F2R protein expression, and blue lines indicate low F2R protein expression.

The survival analysis of F2R expression in HGSOC patients was performed according to H-scores obtained from the HGSOC TMA data. The patients were initially stratified according to H-score quartiles in the Kaplan–Meier survival analysis. We found that patients with an H-score in the lowest 25% quartile had increased progression-free survival (PFS) compared with the other three quartiles. We defined the lowest 25% quartile as low expression, and the other three quartiles (the remaining 75%) were defined as high F2R expression. Statistically significant shorter PFS was observed (Figure 4A, *p* = 0.037), suggesting high F2R expression to be linked to worse patient outcome and highlighting a possible role as a prognostic marker. In contrast, overall survival (OS) seemed reduced up to approximately 3 years but was not statistically significant over longer time points (Figure 4B, *p* = 0.418).

### 2.5. F2R Knockdown in Ovarian Cancer Cell Lines Alters the Tumor Cell Biology

**Figure 5:**
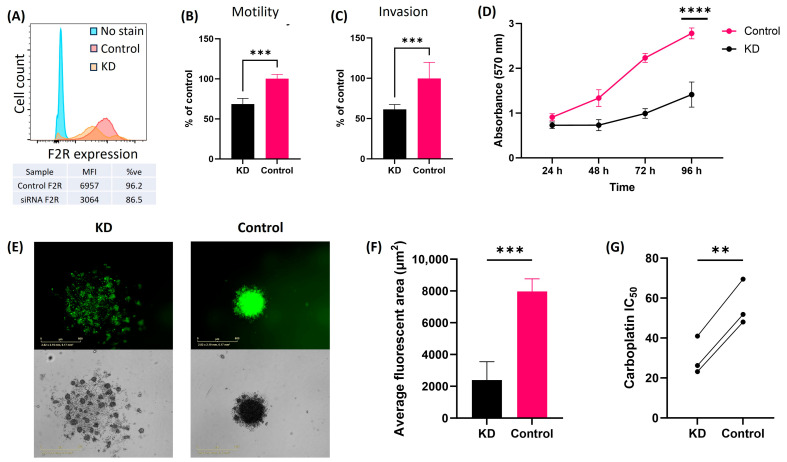
F2R knockdown alters the motility, invasive properties, and metabolic potential of ES-2 cells. (**A**) Flow cytometry analysis of F2R expression comparing scrambled siRNA control and F2R siRNA knockdown in ES-2 cells. (**B**) Motility and (**C**) invasion of ES-2 cells (F2R knockdown (KD) and control) in ChemoTx^®^ motility and invasion assays (*n* = 8 from 3 independent experiments). (**D**) Cell metabolism assay for ES-2 cells with F2R KD (black line) and control (pink line) at 24 h, 48 h, 72 h, and 96 h (*n* = 15 from 3 independent experiments), with absorbance reading at 570 nm. (**E**) Representative image for the spheroid formation at 72 h for calcein-AM-stained ES-2 cells with F2R knockdown (left image) and control (right image) (scale bar 800 µm). (**F**) Quantification of average spheroid area formation for ES-2 cells with KD and control (*n* = 4). (**G**) Carboplatin IC_50_ determined for ES-2 KD and control (*n* = 12 from 3 independent experiments). Statistical analysis includes Mann–Whitney test; **** *p* < 0.0001, *** *p* < 0.001, and ** *p* < 0.01. Data presented as mean ± SD.

ES-2 and SKOV3 ovarian cancer cell lines have been extensively characterized and show high transfection efficiency, making them valuable in vitro models for functional studies with high experimental reproducibility. These cell line models show high F2R gene/protein expression [3] and therefore provide a suitable platform to investigate F2R-mediated cancer mechanisms. While the cell line models have recognized limitations, they do provide some relevant insights for clinical findings and the understanding of F2R biology in ovarian cancer. Using lipofectamine-assisted siRNA transfection, F2R expression was knocked down by siRNA and confirmed using RT-qPCR (Figure S2), and flow cytometry analysis confirmed protein downregulation in F2R siRNA versus scrambled siRNA controls (Figure 5A). Knockdown in ES-2 cells significantly reduced the cell motility (Figure 5B, *p* = 0.0006) and invasion (Figure 5C, *p* = 0.0006) in vitro compared to the scrambled siRNA control.

We assessed the effect of F2R knockdown on cell metabolism using MTT assays. ES-2 cells with F2R knockdown had significantly slower cell metabolism compared to control cells (Figure 5D). Additionally, ES-2 cells following F2R knockdown (Figure 5E, left) lost the ability to form intact spheroids over 72 h when compared to the control cells (Figure 5E, right). A significant difference in the average fluorescent area (µm^2^) was observed in ES-2 cells with F2R knockdown versus control (Figure 5F, *p* = 0.0002). ES-2 F2R KD cells were more sensitive to carboplatin (Figure 5G, IC_50_ 26.2 µM) compared to control cells (IC_50_ 51.8 µM).

**Figure 6:**
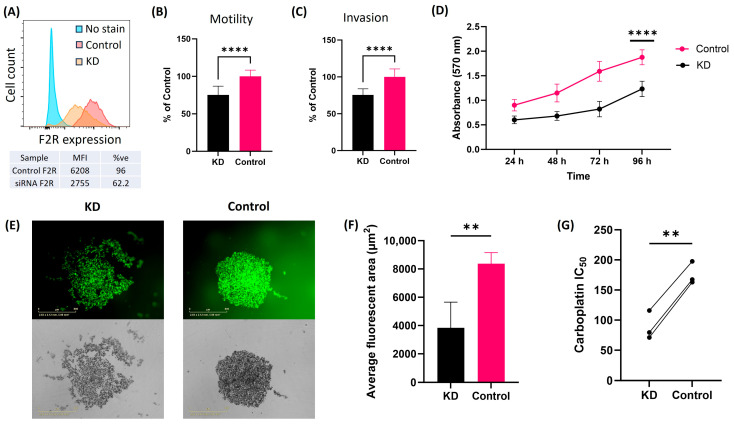
F2R knockdown alters the motility, invasive properties, and metabolic potential of SKOV3 cells. (**A**) Flow cytometry analysis of F2R expression in scrambled siRNA control and F2R siRNA knockdown in SKOV3 cells. (**B**) Motility and (**C**) invasion of SKOV3 cells (F2R knockdown (KD) and control) in ChemoTx^®^ invasion assays (*n* = 12 from three 3 independent experiments). (**D**) Cell metabolism assay for SKOV3 cells with F2R KD (black line) and control (pink line) at 24 h, 48 h, 72 h, and 96 h (*n* = 15 from 3 independent experiment), with absorbance reading at 540 nm. (**E**) Representative image for the spheroid formation at 72 h for calcein-AM-stained SKOV3 cells with F2R knockdown (left image) and control (right image) (scale bar 800 µm). (**F**) Quantification of average spheroid area formation for SKOV3 cells with KD and control (*n* = 4). (**G**) Carboplatin IC_50_ for SKOV3 KD and control cells (*n* = 12 from 3 independent experiments). Statistical analysis includes Mann–Whitney test; **** *p* < 0.0001 and ** *p* < 0.01. Data presented as mean ± SD.

F2R protein expression was reduced using siRNA-mediated gene knockdown in SKOV3 cells, and the knockdown was confirmed using RT-qPCR (Figure S2). In SKOV3 cells, more than a 50% reduction in median fluorescent intensity (MFI) was observed following F2R knockdown to that of the control (Figure 6A). Similar to the results seen with ES-2 cells, F2R knockdown in SKOV3 cells significantly reduced cell motility (Figure 6B, *p* < 0.0001) and invasion (Figure 6C, *p* < 0.0001) compared to that of the scrambled siRNA control.

SKOV3 cells with F2R KD were also observed to have reduced cell metabolism (Figure 6D) over a period of 96 h compared to scrambled siRNA control cells. Furthermore, spheroid formation was less compact in SKOV3 with F2R KD compared to the control (Figure 6E), contributing to the lower fluorescent area size (µm^2^) in the KD cells (Figure 6F, *p* = 0.0038). Like ES-2 cells, SKOV3 cells with F2R KD were more sensitive to carboplatin (IC_50_ 116 µM) compared to control cells (IC_50_ 197.60 µM) (Figure 6G). Additionally, public data from GSE172016 demonstrated significantly high F2R expression in paclitaxel-resistant OVCAR3 and TOV21G cells, further supporting the role of F2R in sensitizing cancer cells to chemotherapy treatments (Figure S3).

## 3. Discussion

Current ovarian cancer biomarkers, such as CA-125 and HE4, lack sensitivity [23]. The development of more effective biomarkers could enhance diagnostic precision, offering prognostic potential and providing novel drug targets that would improve service provision and clinical outcomes. Our study demonstrates consistently high *F2R* gene expression across various ovarian cancer subtypes in both primary and metastatic tissues, positioning F2R as a promising biomarker with diagnostic potential. This has also been validated in one of our previous studies using patient-derived ascites samples and established ovarian cancer cell lines using RT-qPCR [1]. The F2R protein expression observed in both liquid ascites samples and solid tumors directly supports the potential for the development of clinical pathology applications.

The consistent increase in F2R protein expression in solid ovarian cancer tissues and Kaplan–Meier survival analysis demonstrated a correlation between high F2R expression and shorter PFS. This association with poor patient outcomes further confirmed the prognostic potential of F2R as a biomarker. Although the OS did not differ significantly between the high- and low-F2R-expressing groups, this may be due to the additional clinical variables, such as tumor heterogeneity, post-progression therapies, and limited follow-up duration, which can obscure OS differences. Conversely, PFS is frequently a more sensitive indicator of early treatment failure and tumor aggressiveness, making it particularly relevant for biomarker discovery in ovarian cancer. This also aligns with findings in other malignancies, such as breast and pancreatic cancers, where F2R expression correlates with aggressive disease characteristics and reduced survival rates [24,25,26]. We also provided evidence of elevated F2R protein expression in metastatic cancer tissues compared to primary tumors, suggesting its involvement in metastatic disease progression. This might be mechanistically linked to epithelial–mesenchymal transition [27], which is a hallmark of HGSOC [28] and a major contributor to poor prognosis. In breast cancer, F2R also facilitates cell migration and extracellular matrix remodeling, key processes in metastasis that are known to be mediated by PAR protein family members [4,29]. F2R protein expression was also significantly elevated in chemotherapy-resistant ovarian cancer tissues, further underscoring its prognostic significance. Chemotherapy resistance remains a major obstacle in ovarian cancer treatment, often leading to therapeutic failure and disease recurrence. F2R overexpression in chemotherapy-resistant samples may be linked to the activation of survival signaling pathways, such as the Akt and MAPK pathways, both of which are commonly associated with PAR family-mediated mechanisms [30,31]. These findings also suggest that F2R expression could be utilized to stratify patients based on their risk of recurrence and to guide treatment decisions. To further consolidate the F2R protein as a suitable prognostic biomarker, an independent validation cohort study needs to be performed to verify biomarker performance and clinical utility.

The consistent overexpression of F2R, particularly in metastatic and chemotherapy-resistant samples, also highlights its potential as therapeutic target. This was further supported by our in vitro studies demonstrating that siRNA-mediated F2R suppression impaired ovarian cancer cell motility, invasion, and spheroid formation. We acknowledge that the molecular and histological diversity of ovarian cancer is not entirely captured by the cell line models used (SKOV3 and ES-2) despite their well-characterized and experimentally accessible nature. ES-2 is derived from the clear-cell subtype, which has distinct biological features, while SKOV3 does not closely reflect HGSOC. More comprehensive evidence across various ovarian cancer subtypes may be achieved through future research that employs patient-derived organoids or xenograft models. The formation of less compact spheroids and the observed decline in cellular metabolism following F2R knockdown suggest a critical role in sustaining tumor growth within three-dimensional environments, such as ascites or metastatic niches. This aligns with F2R’s known function in regulating cytoskeletal dynamics and facilitating interactions with the extracellular matrix, two essential processes for tumor metastasis [32]. Supporting this hypothesis, a study by Yang et al. demonstrated that F2R overexpression enhanced spheroid formation and self-renewal capacity in human breast cancer cells [33]. Furthermore, F2R downregulation sensitized ovarian cancer cells to carboplatin treatment, indicating its involvement in chemotherapy resistance. F2R, a member of the PAR family, is recognized for its association to many G-protein signaling pathways, particularly Gαq, Gα12/13, and Gαi. These downstream pathways concentrate on critical effectors such as MAPK/ERK, PI3K/AKT, and NF-κB, which govern tumor-associated functions including proliferation, migration, invasion, and survival [34]. In many tumors, PAR1 activation has been demonstrated to facilitate epithelial–mesenchymal transition and matrix remodeling via MAPK and RhoA signaling, whereas PI3K/AKT activation augments chemoresistance and survival [15,26,29,34]. These findings suggest that combining F2R-targeted therapies with platinum-based chemotherapy could enhance treatment efficacy and overcome drug resistance.

As a cell surface receptor belonging to the GPCR family, F2R is well suited for targeted therapies, including small-molecule inhibitors, drug-loaded nanoparticles, or drug-conjugated monoclonal antibodies. The detection of F2R on live cells is an important factor for targeted therapeutics, such as antibody–drug conjugates, which rely on interaction with the cancer cells and need to be internalized to be effective. We provided preliminary evidence of F2R antibody interaction with the surface of ovarian cells and the observation of structures resembling endosomes that were consistent with internalization into live cells. Further investigations are needed to establish cell surface interaction, internalization, endosome delivery and therapeutic killing potential of specific F2R antibody–drug conjugates. Collectively, these findings underscore F2R’s critical role in ovarian cancer pathophysiology and highlight its promise as a novel future therapeutic target.

In summary, our findings suggest that the F2R biomarker could have both diagnostic and prognostic potential, warranting the development of specific assay technology that can be validated and cross-validated to establish accuracy and utility for clinical pathology service provision. While it is rare for biomarkers to have both diagnostic and prognostic applications, there was evidence of increased expression of F2R with disease progression, and we showed that the loss of F2R could impact key aspects of tumor metastasis such as motility, invasion, and metabolic potential. The consistent detection of F2R gene and protein expression in samples from ovarian cancer patients suggests that F2R could be a potential key component of the ovarian cancer pathogenic process and an integral component of the cancer biology. Although these data provide significant groundwork for the translational potential of F2R, it is crucial to recognize that our results originate from preclinical models. The findings from the cohort studies presented here justify further investigation to fully establish F2R as a biomarker. Subsequent clinical translation may be exploited for both the development of novel diagnostic/prognostic technology, and therapeutic treatment.

## 4. Materials and Methods

### 4.1. Materials

ES-2 (RRID: CVCL_3509) and SK-OV-3 (RRID: CVCL_0532) ovarian cancer cell lines were purchased from Sigma-Aldrich (Australia) and American Type Culture Collection (ATCC, Manassas, VA, USA), respectively. Roswell Park Memorial Institute (RPMI 1640), fetal bovine serum (FBS), penicillin–streptomycin (10,000 U/mL), Dulbecco’s phosphate-buffered saline (DPBS), trypsin–ethylenediaminetetraacetic acid, trypan blue solution, primer sequences for real-time quantitative polymerase chain reaction (RT-qPCR), dimethyl sulfoxide (DMSO; ≥99.5%), and 3-(4,5-dimethylthiazol-2-yl)-2,5-diphenyltetrazolium bromide (MTT; ≥98%) were purchased from Sigma-Aldrich (Melbourne, Australia). Advanced RPMI 1640 medium, GlutaMAX^TM^ supplement (100X), eBioscience™ Fixable Viability Dye, calcein-AM, geltrex, countless cell-counting chambers, and lipofectamine™ RNAiMAX transfection reagent were purchased from Thermo Fisher Scientific (Life Technologies, Melbourne, Australia). RNeasy^®^plus mini kits were purchased from Qiagen (Melbourne, Australia). Hard shell^®^ 96-Well PCR plates, Microseal ‘B’ PCR plate sealing film, the iScript^TM^ cDNA synthesis kit, and iTaqTM universal SYBR^®^ green supermix were purchased from Bio-Rad (Adelaide, Australia). ChemoTx^®^ transwell 96 well-plates were purchased from Neuro Probe (Cotati, CA, USA). Primary polyclonal F2R antibody (26366-1-AP) was purchased from United Bioresearch (Sydney, Australia). Secondary fluorescein isothiocyanate (FITC)-labeled goat anti-rabbit IgG (H+L) antibody was purchased from Abclonal via Genesearch (Gold Coast, Australia). The Cytofix/Cytoperm kit was purchased from BD Biosciences (San Jose, CA, USA). Carboplatin (450 mg/45 mL) was obtained from Hospira (Adelaide, Australia).

### 4.2. Public Data

The TNM Plot database (https://tnmplot.com/analysis/) (accessed on 1 October 2024) was used to compare the average *F2R* expression in normal (*n* = 133) and ovarian cancer tissues (*n* = 374) based on the RNA sequencing data from The Cancer Genome Atlas (TCGA) [21].

Microarray data generated with the GPL570 platform (HG-U133) was analyzed using the GENT2 (http://gent2.appex.kr) (accessed on 14 October 2024) database to assess the *F2R* expression in ovarian cancer (*n* = 1626 patients from 35 genomic spatial event (GSE) datasets) [20]. Patient samples without sufficient histologic characterization data were excluded. The remaining samples were stratified into normal ovarian surface epithelium (OSE) (*n* = 66), fallopian tube (FT) (*n* = 40), epithelial ovarian (*n* = 1162), or metastatic tumor (*n* = 10). Most of the ovarian epithelial tumors (*n* = 1054) were classified as either low-grade serous ovarian cancer (LGSOC) (*n* = 41), high-grade serous ovarian cancer (HGSOC) (*n* = 806), clear-cell ovarian cancer (*n* = 77), mucinous ovarian cancer (*n* = 32), or endometroid ovarian cancer (*n* = 98). The GSE40595 dataset was used to analyze laser micro-dissected HGSOC tumors for *F2R* expression in matching stroma and epithelial tissues (*n* = 28). An overview of the used public data is in the Supplementary Information (SI), Table S1. *F2R* (probe: 203989_x_ at) expression was also analyzed in control and paclitaxel-resistant ovarian cancer cell lines (OVCAR3 and TOV21G, GSE172016) using the NCBI GEO2R (https://www.ncbi.nlm.nih.gov/geo/geo2r/) (accessed on 20 November 2024) database.

### 4.3. Patient Tissue Cohorts

Samples of normal ovary, FT, and HGSOC tissues were collected at the Royal Adelaide Hospital (RAH) following human ethics guidelines (RAH protocol numbers 140101, HREC/14/RAH/13, and 060903 (primary and metastatic tissue microarray (TMA) cohort)). Paraffin-embedded tissue slides were prepared for normal FT (*n* = 11), normal ovaries (*n* = 10), and benign ovarian tissues (*n* = 10) (Table S2) diagnosed between 2010 and 2019, according to published protocols [35]. Paraffin-embedded HGSOC patient samples, including primary (*n* = 83), metastatic (*n* = 35) (Table S3), and chemotherapy-sensitive (*n* = 19) and -resistant (*n* = 19) (Table S4) tissues were also used to assemble TMA slides. Patients were classified as chemotherapy sensitive and resistant as described previously [36]. Patients who exhibited complete response and did not progress within 6 months of chemotherapy treatment completion were classified as sensitive, while those patients that did not respond or relapsed within 6 months following treatment were considered resistant.

### 4.4. Immunohistochemistry

Immunohistochemistry (IHC) was performed as previously described, using microwave antigen retrieval followed by overnight incubation at 4 °C using the F2R rabbit polyclonal antibody (1 in 750 dilution) [35,36]. Biotinylated anti-rabbit immunoglobulins, streptavidin–peroxidase conjugate, and diaminobenzidine substrate were used to visualize the immunoreactivity [35]. For negative controls, tissues were incubated without primary antibody. The slides were digitally scanned using the NanoZoomer Digital Pathology System (Hamamatsu Photonics, Hamamatsu city, Japan). QuPath software (version 0.5.1) was used to quantify F2R immunostaining in the cancerous/epithelial tissues [37]. The average F2R H-scores were evaluated for each sample.

### 4.5. Cell Culture and Patient-Derived Samples

The Mycoalert detection kit (Lonza, Sydney, Australia) was used to confirm that all cell cultures were mycoplasma negative. The ES-2 and SK-OV-3 cell lines were maintained in RPMI medium with 1% (*v*/*v*) penicillin–streptomycin and 10% (*v*/*v*) FBS. Cells were cultured at 37 °C in 5% CO_2_ environment until cell confluency was at least 90%.

ES-2 cells were authenticated on 12 April 2021 using short tandem repeat analysis, with the Promega GenePrint^®^ 10 system (Griffith University DNA sequencing facility, Brisbane, Australia). SK-OV-3 cells were authenticated by the Australian Genome Research Facility (AGRF) (Adelaide, Australia) on 14 October 2022.

The clinical details of the patient primary cells (approved by the RAH Human Research Ethics Committee HREC/14/RAH/13 and HREC/18/CALHN/811, with patient informed consent) are shown in Table S5. Advanced RPMI medium supplemented with 10% (*v*/*v*) FBS, 1% (*v*/*v*) penicillin–streptomycin, and 1% (*v*/*v*) GlutaMAX^TM^ was used to culture the primary ovarian cancer cells. Cells were maintained at 37 °C and 5% CO_2_ as described previously [36].

### 4.6. Flow Cytometry

Single cells were resuspended in PBS and stained with a live/dead marker in PBS (1 in 3000 dilution) for 20 min at 4 °C. Cells were then washed, fixed, and permeabilized using the BD Cytofix/Cytoperm kit, as per the manufacturer’s instructions. Cells were then stained with primary antibody (1:100 dilution in permeabilization buffer, 30 min at 4 °C incubation), washed, stained with secondary antibody 1:100 dilution in permeabilization buffer, 30 min at 4 °C incubation), and resuspended in fluorescence-activated cell sorting (FACS) buffer (PBS + 2% FBS) prior to running the samples on the FACSAriaTM Fusion flow cytometer (BD Biosciences, San Jose, CA, USA). Data were analyzed using FlowJoTM software (Version 10, BD Biosciences, San Jose, CA, USA). Figure S1 presents the gating strategy for F2R quantification.

### 4.7. Immunofluorescence

To detect F2R at the cell surface, ascites-derived cells were seeded (5000 cells) on coverslips for 72 h, probed with primary antibody for 1 h, followed by anti-rabbit AlexaFluor-conjugated secondary antibody (1:1000, #A31573, Thermo Fisher, Melbourne, Australia) and Hoechst stain (1:1000, #33342; Thermo Fisher, Melbourne, Australia) for 30 min on ice. Cells were finally fixed in a solution containing 4% paraformaldehyde (Emgrid, Adelaide, Australia) and 4% sucrose in PBS for 8 min, and the coverslips were mounted using ProLong™ Gold Antifade Mountant (Thermo Fisher Scientific, Melbourne, Australia).

A Nikon A1+ confocal microscope (Nikon, Tokyo, Japan) with a LU-N4/LU-N4S 4-laser unit (403, 488, 561, and 638 nm) and a Plan Apo λ 60× oil-immersion objective lens (1.4 N.A.) at 1.2 AU pinhole was used for fluorescence microscopic imaging. NIS Elements software (v4.5, Nikon) was used for this purpose. The Galvano GaAsP detector was used for imaging at 512 pixels, with a piezo z-stage, 2× line averaging, 1× zoom (0.42 μm/px), and z-steps of 0.4 μm.

### 4.8. siRNA Gene Knockdown and RT-qPCR

Three sets of siRNAs, including scrambled siRNA as a control (GenePharma, Shanghai, China, Table S6), were tested for F2R knockdown in both ES-2 and SK-OV-3 cell lines. The lipofectamine RNAiMax transfection agent was used according to the manufacturer’s instructions to deliver 25 picomoles of siRNA per well into 6-well plates, followed by 48 h incubation. RNA was isolated from the transfected cells using a RNeasy^®^plus mini kit. A NanoDrop 1000 spectrophotometer (Thermo Fisher Scientific, Melbourne, Australia) was used to analyze RNA quality and concentration, and cDNA was produced using the iScript^TM^ cDNA synthesis kit. F2R mRNA expression was assessed using an RT-qPCR method as mentioned previously [3], and the human housekeeping gene succinate dehydrogenase complex flavoprotein subunit A (SDHA) was used as an internal control.

### 4.9. Motility and Invasion Assay

Cell motility and invasion tests were carried out as previously reported using 40,000 cells/well in a 96-well microtiter plate [38]. In brief, ES-2 or SK-OV-3 cells (with or without *F2R* gene knockdown) were labeled with calcein-AM (1 µg/mL, Invitrogen, Adelaide, Australia) for 30 min, followed by washes to remove excess calcein-AM, and loaded onto either 12 µm filters coated with Geltrex (0.6 µL/well, Invitrogen, Adelaide, Australia) for invasion tests or uncoated 12 µm filter inserts (96-well plate, ChemoTx, Neuro Probe, Cotati, CA, USA) for migration studies. A Triad series multimode detector (Dynex Technologies, Chantilly, VA, USA) was used in bottom-read fluorescence mode (485–520 nm) to monitor cells that migrated or invaded the lower chamber with chemoattractant after 6 h of incubation.

### 4.10. Cell Metabolism Assay

The MTT assay was used to measure the cell metabolism of control and knockdown samples in 96-well plates seeding 2000 and 3000 ES-2 and SK-OV-3 cells/well, respectively. After 24 h, 48 h, 72 h, or 96 h of incubation, the cells were treated with MTT solution in medium (0.5 mg/mL, 100 µL/well), followed by incubation for 4 h at 37 °C. Then, 150 µL of DMSO was added to each well, and a multimode plate reader (VICTOR^®^ Nivo™ Plate Readers, Perkin Elmer, Melbourne, Australia) was used to read the absorbance at 570 nm.

### 4.11. Carboplatin Response Assay

For control and knockdown samples, ES-2 and SK-OV-3 cells were seeded at 2500 and 3000 cells/well, respectively. After 24 h of cell culture, the cells were treated with serial 1:2 dilutions of carboplatin (3000–3 μM) prepared in growth media. Cells were incubated for an additional 72 h prior to MTT analysis. The IC_50_ values of carboplatin were determined for both cell lines using GraphPad Prism software (Version 10.1.2).

### 4.12. Spheroid Assay

Calcein-AM-stained knockdown and control samples of ES-2 and SK-OV-3 cells were plated at 10,000 cells/well in 96-well Corning^®^ Costar ^®^ Ultra-Low Attachment Plates (CLS7007). Images of spheroid formation were taken in real time, every 6 h for 3 d, using the Sartorius Incucyte^®^ SX5 Live Cell Analysis System (Adelaide Microscopy, University of Adelaide, Adelaide, Australia) and the green fluorescence channel. Sizes of individual spheroids were assessed with the Incucyte^®^ Spheroid Analysis Software (v2025B) Module adaptive segmentation method, applying a threshold adjustment of five. Data were presented as an average of the green object area (µm^2^).

### 4.13. Statistical Analysis

One-way ANOVA analysis and the unpaired Student’s T test were used for normally distributed data in cell line studies and online database analysis (GraphPad Prism, Version 10.1.2, San Diego, CA, USA). Normality for each dataset was assessed using the Shapiro–Wilk test. For data with a non-normal distribution, the Kruskal–Wallis test along with Dunn’s multiple comparison test, Wilcoxon rank paired test, or Mann–Whitney test was used. At *p* < 0.05, statistical significance was recognized. Significance ranking is indicated throughout this report as follows: * = *p* < 0.05, ** = *p* < 0.01, *** = *p* < 0.001, **** = *p* < 0.0001, and non-significant (ns). KM survival analysis for the TMA cohort was performed using IBM^®^ SPSS^®^ Statistics software, and the log-rank test was used to evaluate the differences in survival probabilities (Version 28.0.1.0, IBM^®^ Corporation, Armonk, NY, USA).

## 5. Conclusions

F2R represents a promising candidate for addressing key challenges in ovarian cancer diagnosis, prognosis, and treatment. Its elevated expression in tumor tissues, particularly in metastatic and chemotherapy-resistant samples, highlights its potential as a diagnostic/prognostic biomarker and a therapeutic target. By integrating F2R-targeted therapies into precision oncology frameworks, it may be possible to improve early detection, stratify patients based on recurrence risk, and enhance treatment efficacy. Future research should focus on validating F2R’s clinical utility and developing innovative therapeutic strategies to leverage its unique role in ovarian cancer progression.

To establish F2R as a clinically viable biomarker and therapeutic target, several steps are necessary. First, large-scale validation studies should assess its diagnostic and prognostic performance in diverse patient cohorts. Second, mechanistic studies are needed to elucidate the molecular pathways through which F2R drives metastasis and chemoresistance. Finally, preclinical studies should evaluate the efficacy of F2R-targeted therapies, including small-molecule inhibitors, monoclonal antibodies, and nanomedicines in combination with standard chemotherapy. Additionally, epitope mapping and antibody engineering are essential for developing specific immunochemical reagents to enhance F2R detection, prognosis, and targeted therapy.

One potential application for F2R is the development of a standardized IHC-based prognostic test to convert it into a clinically useful biomarker. This would involve the use of formalin-fixed, paraffin-embedded tumor samples to assess F2R expression using a semi-quantitative method, such as H-score. Patients could be categorized as low or high F2R expressers to facilitate treatment planning, such as identifying those at a higher risk of early progression or chemotherapy resistance. A standardized ELISA or immunoassay approach could also be established for quantifying circulating F2R levels in patient serum or plasma for diagnostic purposes. A test of this nature could be a valuable addition to current clinical markers, such as CA-125, and could be particularly beneficial for the selection of patients who may benefit from F2R-targeted therapies or inclusion in theranostic trials. In order to validate thresholds and evaluate their clinical utility in routine pathology settings, additional prospective studies are required.

## Data Availability

The datasets used and/or analyzed during the current study are available from the corresponding authors on reasonable request.

## Supplementary Materials

The following supporting information can be downloaded at: https://www.mdpi.com/article/10.3390/ijms26178529/s1.

## Abbreviations

The following abbreviations are used in this manuscript:

AGRF	Australian Genome Research Facility
ATCC	American Type Culture Collection
DMSO	Dimethylsulfoxide
DPBS	Dulbecco’s phosphate-buffered saline
ERK	Extracellular signal-regulated kinase
F2R	Coagulation factor II receptor
FACS	Fluorescence-activated cell sorting
FBS	Fetal bovine serum
FITC	Fluorescein isothiocyanate
FT	Fallopian tube
GPCRs	G-protein coupled receptors
GSEs	Genomic spatial events
HGSOC	High-grade serous ovarian cancer
IHC	Immunohistochemistry
KM Plot	Kaplan–Meier plot
LGSOC	Low-grade serous ovarian cancer
MMPs	Matrix metalloproteinases
MTT	3-(4,5-dimethylthiazol-2-yl)-2,5-diphenyltetrazolium bromide
ns	Non-significant
OS	Overall survival
OSE	Ovarian surface epithelium
PAR1	Protease-activated receptor 1
PARs	Protease-activated receptors
PFS	Progression-free survival
RAH	Royal Adelaide Hospital
Rap1/MAPK	Ras-associated protein 1/Mitogen-activated protein kinase
RPMI	Roswell Park Memorial Institute
RT-qPCR	Real-time quantitative polymerase chain reaction
SDHA	Succinate dehydrogenase complex flavoprotein subunit A
TCGA	The Cancer Genome Atlas
TMA	Tissue microarray
