# Supplementary material for: Protease-Activated Receptor F2R Is a Potential Target for New Diagnostic/Prognostic and Treatment Applications for Patients with Ovarian Cancer
Source: Int J Mol Sci. 2025 Sep 2;26(17):8529. doi: 10.3390/ijms26178529 (PMC12428987; doi:10.3390/ijms26178529)
Supplement: Supplementary file 1 [file ijms-26-08529-s001.zip › ijms-3772753-supplementary.pdf]

Supplementary information

Protease-Activated Receptor F2R Is a Potential Target for New Diagnostic/Prognostic and Treatment Applications for Patients with Ovarian Cancer

Riya Khetan [1], Noor A. Lokman [2], Preethi Eldi [3], Zoe K. Price [2], Martin K. Oehler [2,4], Doug A. Brooks [3], Anton Blencowe [1,5], Sanjay Garg [1], Carmela Ricciardelli [2,*] and Hugo Albrecht [1,*]

1 Centre of Pharmaceutical Innovation, UniSA Clinical and Health Sciences, University of South Australia, Adelaide, SA 5000, Australia; riya.khetan@mymail.unisa.edu.au (R.K.); anton.blencowe@unisa.edu.au (A.B.); sanjay.garg@unisa.edu.au (S.G.)

2 Discipline of Obstetrics and Gynaecology, Adelaide Medical School, Robinson Research Institute, University of Adelaide, Adelaide, SA 5000, Australia; noor.lokman@adelaide.edu.au (N.A.L.); zoe.price@adelaide.edu.au (Z.K.P.); martin.oehler@adelaide.edu.au (M.K.O.)

3 UniSA Clinical and Health Sciences, University of South Australia, Adelaide, SA 5000, Australia; preethi.eldi@unisa.edu.au (P.E.); doug.brooks@unisa.edu.au (D.A.B.)

4 Department of Gynaecological Oncology, Royal Adelaide Hospital, Adelaide, SA 5000, Australia

5 Applied Chemistry and Translational Biomaterials Group, Centre of Pharmaceutical Innovation, UniSA Clinical and Health Sciences, University of South Australia, Adelaide, SA 5000, Australia

* Correspondence: carmela.ricciardelli@adelaide.edu.au (C.R.); hugo.albrecht@unisa.edu.au (H.A.)

Table S1. Overview of public data sets.

	Minimum	25% percentile	Median	75% percentile	Maximum
**TNM Plot**					
Normal (n=133)	27	78	127	238	869
Tumour (n=374)	44	268	457	847.8	3707
**GENT2**					
OSE (n=66)	4.17	5.888	6.714	7.197	10.5
FT (n=40)	1.585	2.585	3.683	6.655	7.687
Cancer (n=1,162)	2.807	6.867	7.562	8.254	10.65
Metastatic (n=10)	5.672	7.064	7.835	8.2	8.855
**Subtypes**					
LGSOC (n=41)	4.459	6.451	6.989	7.512	9.646
HGSOC (n=806)	2.807	6.778	7.539	8.18	10.35
Clear cell (n=77)	6.044	7.281	8.017	8.527	10.65
Mucinous (n=32)	6.524	7.103	7.687	8.16	9.572
Endometroid (n=98)	5.426	7.085	7.714	8.524	10.08
**GSE40595**					
Stroma (n=28)	6.322	7.324	7.908	8.594	10.57
Epithelium (n=28)	6.615	7.221	7.71	8.371	9.16

Table S2. Patient information for immunohistochemistry slides prepared using normal fallopian tube, ovaries, and benign ovarian cancer tissues.

Patient Cohort	Diagnosis	n	Age
**Normal ovaries (n = 10)**	Menorrhagia, normal ovaries	2	49, 51
	Fibroid uterus	1	55
	Endometrial polyp, normal control	1	76
	Fibroid uterus, normal control	1	46
	BRCA1, pHx breast ca, normal adnexa, and uterus in histopathology	1	41
	BRCA1 mutation carrier, normal histopath of ovaries	2	46, 62
	Elevated oestrogen, no pathology, normal control	1	43
	Family history of ovarian cancer, normal uterus, ovaries	1	51
**Normal tube (n = 11)**	Fibroid uterus	1	55
	Benign bulky fibroid uterus	1	58
	Benign tube and ovary	1	58
	Benign endometrial polyp	1	67
	Benign serous cystadenoma of the ovary	1	60
	Menorrhagia, normal uterus and adnexa, normal control	3	39, 48, 49
	BRCA1 mutation carrier, normal histopath of tubes and ovaries	2	37, 46
	Endometrial hyperplasia with atypia	1	64
**Serous benign tumour (n = 10)**	Benign serous cystadenoma of the ovary	7	25-76
	Benign serous cystadenofibroma of the ovary	3	57-80

Table S3. Clinicopathological features of the HGSOC TMA cohort and the tissues of corresponding HGSOC patients.

**Primary serous carcinomas (n=83)**		
**Age at Diagnosis (years)**	Median (range)	59 (24-86)
**Histological Grade**	Grade 2	15
	Grade 3	68
**FIGO stage**	Stage II	1
	Stage III	80
	Stage IV	1
**F2R, H-score max percentiles**	25	70
	50	100
	75	143.6
**Recurrence**	No	17
	Yes	59
	Unknown	7
**Cause of Death**	Ovarian cancer	56
	Other cause	9
	Alive	16
	No follow-up	2
**Metastatic serous carcinomas (n=35)**		
**Age at Diagnosis (years)**	Median (range)	68 (46-86)
**Histological Grade**	Grade 2	6
	Grade3	29
**FIGO stage**	Stage III	33
	Stage IV	2
**F2R, H-score max percentiles**	25	78.7
	50	105.3
	75	158.9
**Recurrence**	No	10
	Yes	20

	Unknown	5
**Cause of Death**	Ovarian cancer	23
	Other cause	2
	Alive	10

Table S4. Patient information for immunohistochemistry tissue microarray (TMA) slides prepared using chemo-sensitive and chemo-resistant tissues (Grade 3).

Patient Cohort	Diagnosis	n	Age
**Chemo-resistant tissues (n = 19)**	Serous peritoneal cancer, Stage 3c	2	48, 78
	Serous ovarian cancer, Stage 2a	1	84
	Serous ovarian cancer, Stage 3b	1	82
	Serous ovarian cancer, Stage 3c	2	57, 66
	Serous papillary carcinoma of the ovary, stage 2c	2	59, 63
	Serous papillary carcinoma of the ovary, stage 3a	2	69, 78
	Serous papillary carcinoma of the ovary, stage 3b	1	49
	Serous papillary carcinoma of the ovary, stage 3c	5	46-78
	Serous papillary carcinoma of the peritoneum, stage 3c	2	51, 55
	Serous papillary carcinoma of the peritoneum, stage 4	1	69
**Chemo-sensitive tissues (n = 19)**	Serous peritoneal cancer, Stage 3c	2	43, 79
	Serous ovarian cancer, Stage 2a	1	53
	Serous ovarian cancer, Stage 3b	1	65
	Serous ovarian cancer, Stage 3c	7	44-69
	High grade serous carcinoma of the ovary, stage 4	2	65, 66
	Serous papillary carcinoma of the ovary, stage 3c	6	59-80

Table S5. Clinical information of patient-derived primary cells.

No.	Age at diagnosis	Diagnosis	Stage at diagnosis	Grade
**P1**	66	Serous carcinoma of the peritoneum	4	3
**P2**	73	Serous carcinoma of the peritoneum	4	3
**P3**	47	Recurrent serous carcinoma of the ovary	1c	3
**P4**	59	Serous carcinoma of the ovary	3	3
**P5**	70	Serous carcinoma of the ovary	3b	3

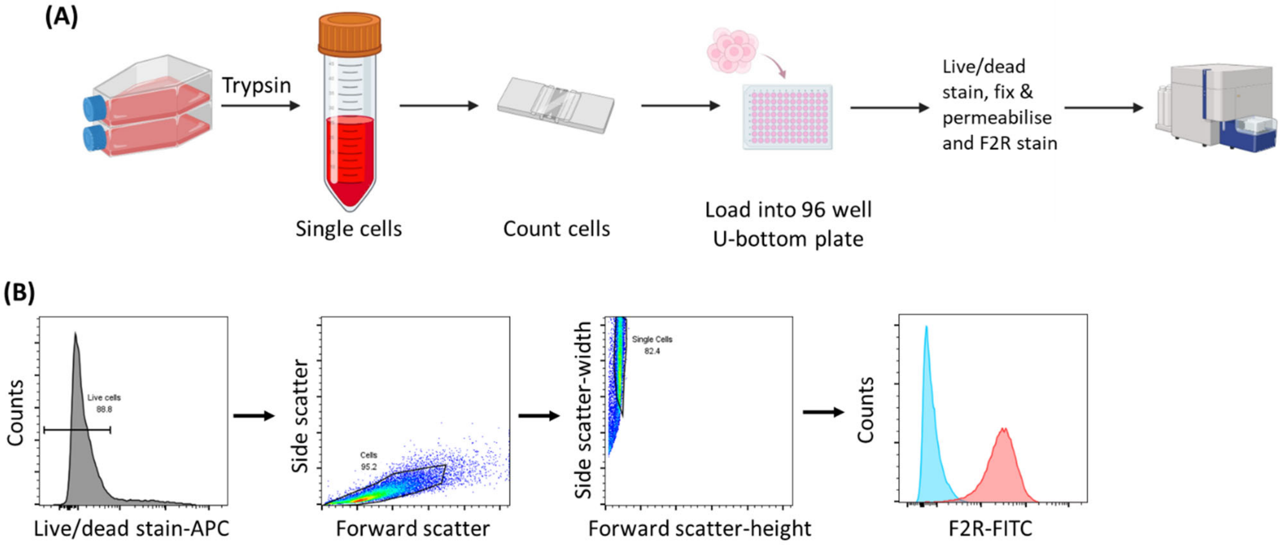

Figure S1. Schematic representation of sample preparation and gating strategy for flow cytometry: A) Steps involved before flow cytometry analysis including cell culture, cell counting, staining, fixing and permeabilisation; B) gating method to quantify cell counts and the last plot depicts two distinct peaks representing unstained cells, and primary plus secondary antibody (FITC) stain for F2R detection. Figure was partially created using BioRender.com.

Table S6. Three sets of siRNAs designed for *F2R* knockdown using Genscript siRNA target finder tool (https://www.genscript.com/tools/sirna-target-finder) followed by blast validation through NCBI.

siRNA set	Sense (5′– 3′)	Antisense (5′– 3′)
**F2R (1)_siRNA**	GUCCUCUAGUGAAUGUAGGTT	CCUACAUUCACUAGAGGACTT
**F2R (2)_siRNA**	GCAACAAAUGCCACCUUAGTT	CUAAGGUGGCAUUUGUUGCTT
**F2R (3)_siRNA**	CAGGACGAGAUGACGGUGUTT	ACACCGUCAUCUCGUCCUGTT

Table S7. Nucleotide sequences of RT-qPCR primers for both *F2R* and *SDHA*. Primers were designed using the NCBI primer design tool.

Receptor name	Forward primer	Reverse primer
SDHA	CTACGACACCGTGAAGGGCT	TGTCCACCAAATGCACGCTG
F2R	AGAGAGGGTGAAGCGGAGCA	TGACCGGGGATCTAAGGTGG

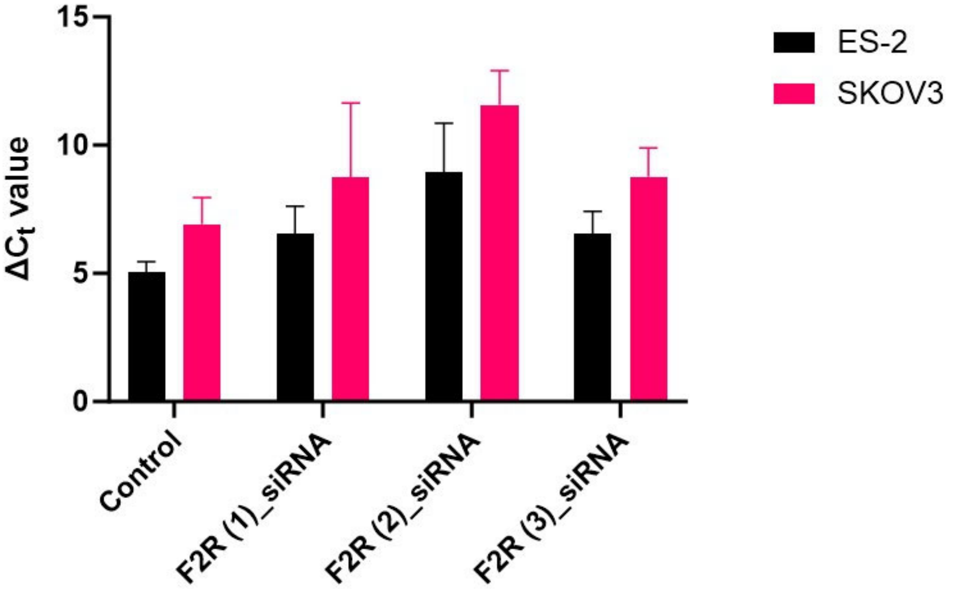

Figure S2. RT-qPCR confirming the F2R knockdown using three sets of siRNAs and SDHA as the housekeeping gene. Y-axis represents the ΔC_t values (C_t(SDHA) – C_t(F2R)) and x-axis represents control (scramble siRNA) along with three siRNA sets for ES-2 and SKOV3. F2R (2)_siRNA was selected for functional assays based on the higher ΔC_t values. Data represents two biological replicates with four samples each.

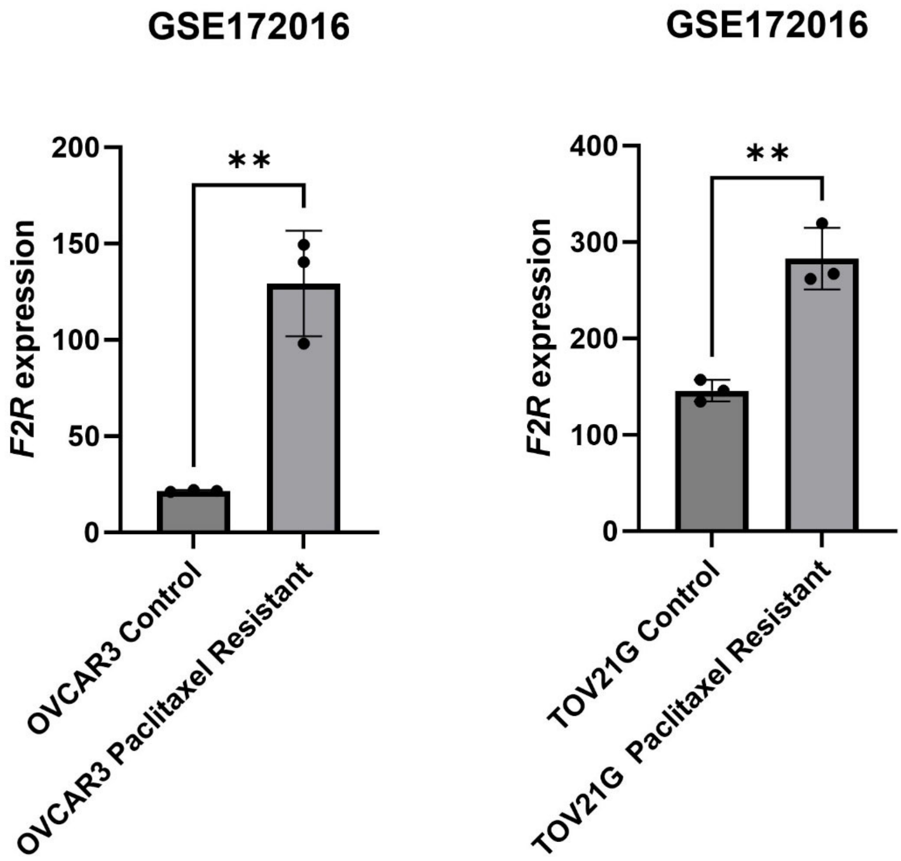

Figure S3. *F2R* expression in OVCAR3 (left) and TOV21G (right) control and paclitaxel resistant ovarian cancer cell lines. Data obtained from publicly available GSE172016 dataset. **p<0.01, unpaired t-test.
